# Metabolic rate of the red panda, *Ailurus fulgens*, a dietary bamboo specialist

**DOI:** 10.1371/journal.pone.0173274

**Published:** 2017-03-17

**Authors:** Yuxiang Fei, Rong Hou, James R. Spotila, Frank V. Paladino, Dunwu Qi, Zhihe Zhang

**Affiliations:** 1 Department of Biodiversity, Earth and Environmental Science, Drexel University, Philadelphia, PA United States of America; 2 Sichuan Key Laboratory of Conservation Biology for Endangered Wildlife, Chengdu Research Base of Giant Panda Breeding, Northern Suburb, FuTou Shan, Chengdu, Sichuan Province, People’s Republic of China; 3 Department of Biology, Indiana Purdue University at Fort Wayne, 2101 E. Coliseum Blvd, Fort Wayne, IN United States of America; Sichuan University, CHINA

## Abstract

The red panda (*Ailurus fulgens*) has a similar diet, primarily bamboo, and shares the same habitat as the giant panda, *Ailuropoda melanoleuca*. There are considerable efforts underway to understand the ecology of the red panda and to increase its populations in natural reserves. Yet it is difficult to design an effective strategy for red panda reintroduction if we do not understand its basic biology. Here we report the resting metabolic rate of the red panda and find that it is higher than previously measured on animals from a zoo. The resting metabolic rate was 0.290 ml/g/h (range 0.204–0.342) in summer and 0.361 ml/g/h in winter (range 0.331–0.406), with a statistically significant difference due to season and test temperature. Temperatures in summer were probably within the thermal neutral zone for metabolism but winter temperatures were below the thermal neutral zone. There was no difference in metabolic rate between male and female red pandas and no difference due to mass. Our values for metabolic rate were much higher than those measured by McNab for 2 red pandas from a zoo. The larger sample size (17), more natural conditions at the Panda Base and improved accuracy of the metabolic instruments provided more accurate metabolism measurements. Contrary to our expectations based on their low quality bamboo diet, the metabolic rates of red pandas were similar to mammals of the same size. Based on their metabolic rates red pandas would not be limited by their food supply in natural reserves.

## Introduction

The red panda (*Ailurus fulgens*) (Cuvier, 1825) is called a “panda”, but molecular and chromosomal data place it in its own family, Ailuridae. It is related to weasels, otters, raccoons, kinkajous and skunks that are all members of the superfamily Musteloidea [1, 2]. The red panda has a similar diet, primarily bamboo, and shares the same habitat as the giant panda, *Ailuropoda melanoleuca* [3]. The false thumb (carpal bone) of the red panda evolved as an adaptation to climbing and secondarily developed for item manipulation. Thus, the red panda’s adaptation for eating bamboo is a case of convergent evolution with the giant panda [4]. Ecological studies of the red panda have defined its home range and seasonal activity in different nature reserves [3, 5, 6] and described habitat use and separation between the red panda and the giant panda [3]. Threats to its survival and conservation have been known for some time [7]. However, there appears to be a high level of genetic diversity in the red panda populations in Sichuan and Yunnan Provinces [8].

There are considerable efforts underway to understand the ecology of the red panda and to increase its populations in natural reserves. Yet it is difficult to design an effective strategy for red panda reintroduction if we do not understand its basic biology. Surprisingly there are few studies on the physiology of the red panda. In addition, we do not know the thermoregulatory adaptations that allow this animal to function in the mountains at 2800–3000 m during winter and summer. Ultimately what is needed is knowledge of its biophysical ecology and climate space [9]. The basis for such analyses is information on the metabolic rate of the red panda.

In one of a series of classic studies McNab (1988) [10] measured the resting metabolic rates (RMR) of two red pandas from a zoo. He found that red pandas decreased metabolic rate at low environmental temperatures without reduced body temperature. However, the metabolic rates that McNab measured were much below those predicted by allometric equations of metabolic rate vs. body size in mammals [11]. Therefore, we measured the RMR of red pandas during winter and summer at the Chengdu Research Base of Giant Panda Breeding (Panda Base) in Sichuan Province, China to obtain information on animals that were living under more natural conditions than in McNab’s study.

In addition to the obvious need to accurately measure metabolic rates for understanding the ecology of the red panda, the study of vertebrate resting metabolism has produced important insights into the role of body size in the biology of animals and has led to continuing controversy about the scaling relationship between body size and metabolic rate [11, 12]. It has also led to development of the metabolic theory of ecology (MTE) that aims to provide mathematical equations for the mechanistic underpinnings of ecology [13, 14] relating how body size and temperature, through their effect on metabolic rate, affect rates and timing of ecological processes. However, the MTE has been controversial and there are continuing discussions of the proper scaling exponent in the allometric equation of body size vs. basal metabolic rate (BMR = aM^b^) in mammals where M is body mass [12, 15]. One of the problems in this discussion has been a question about the accuracy of data for some species. Therefore, it is important to obtain accurate measurements of metabolic rate in the red panda because it is a unique species and its metabolic rate can have an important impact on phylogenetic interpretations of allometric equations of body mass vs BMR in mammals.

## Methods

### Red panda acquisition and maintenance

We studied red pandas at the Panda Base in Chengdu, China (www.panda.org.cn) and conducted all experiments in cooperation with the research, veterinary and husbandry staff there. Red pandas lived in their normal enclosures with access to large outside areas and ate a diet composed primarily of bamboo supplemented with foods such as apples and “panda cake”, a biscuit made of a mixture of grains with vitamins. We transported red pandas to the laboratory for each experiment. Animals did not eat for 24 h before the experiment.

This study was approved by the Chengdu Research Base of Giant Panda Breeding and the Institutional Animal Care and Use Committee of Drexel University (Protocol #20032). Permission to work at the Panda Base was given by the Director after consultation with the Research, Husbandry and Veterinary Departments. There was no animal care and use committee at the Panda Base. Instead each of the three departments involved reviewed the protocol and approved it. Then overall approval came from the Director.

### Metabolism experiments

We measured metabolic rate during two seasons, summer and winter. Because there was no effective air temperature-control room at the Panda Base we used natural air temperature change during the seasons to study the red pandas under warm and cool conditions. We did that to assess the thermal neutral zone of the red panda.

We studied 10 red pandas in summer and 7 red pandas in winter. Because red pandas are diurnal, we conducted all experiments during night hours (2130–0500). Red pandas were weighed before and after each experiment. We measured metabolic rate in a Plexiglas® chamber using a flow through system to measure oxygen consumption and carbon dioxide production. The chamber was 1 m * 1 m * 1 m and constructed of 2.0 cm Plexiglas® with a steel frame for added strength. One side of the chamber was a door held by steel hinges, sealed with a rubber gasket and closed with metal latches. There were three 2.5 cm holes, with 60 cm long tubing attached to avoid backflow, for air intake at the bottom right side of the chamber. There was one 2.5 cm exit hole at the top left side of the chamber that connected to spiral-wound tubing leading to a Flowkit -500 mass flow system (Sable Systems International). A subsample of air went from the Flowkit pump to a FOXBOX oxygen and carbon dioxide analyzer (Sable Systems International). The three air intake holes and one air exit hole eliminated negative pressure in the system. The placement of the holes reduced air stagnation and two small battery operated fans in the chamber assured that the air was well mixed. Six 24-gauge Cu-Co thermocouples (+/- 0.05°C) located inside the chamber on the top, right side, left side, back side, and in the mouth of the air intake and exit holes measured chamber temperatures.

The Sable System Flowkit used a precision mass flow sensor with a rotary pump controlled by a microprocessor to control air flow rate to within 2% of reading. The Flowkit pump’s air flow was set at 25 L/min. That flow rate ensured that the air was replaced in the chamber every 30 to 40 minutes. Each experiment lasted between 7 and 8 hours and we recorded data every 20 to 25 min. Red pandas were generally asleep. If an animal was active for a short period of time we could see that effect as an increase in CO_2_ and decrease in O_2_ values during the next reading. Oxygen levels in the chamber generally dropped to 20.53–20.77% and CO_2_ levels rose to 0.14–0.17%. We took the lowest stable readings as the O_2_ and CO_2_ values to calculate metabolic rate. After leaving the Flowkit pump, air was subsampled though a small plastic tube and drawn into the FOXBOX system at a rate of 200 ml/min. The subsample went through a relative humidity meter and temperature meter before it entered the gas analyzers. Sample air passed through the CO_2_ analyzer and then a drierite column before entering the O_2_ analyzer. Since water vapor would interfere with the fuel cell in the oxygen analyzer we removed the water before it entered that analyzer. The accuracy of the Sable System Foxbox was 0.1% for O_2_ over a range of 2–100% and 1.0% of span for CO_2_ over a range of 0–5%. It automatically gave readings of gas values at standard temperature and pressure (STPD). We used calibration gas (14.93% O_2_, 3.99% CO_2_) from Dalian Special Gas Industry Company and tested by National Institute of Measurement and Testing Technology, 100% dry N_2_ and room air to calibrate the system.

### Statistical analysis

We fit a linear mixed effects model (LMER) using the statistical software R (R Development Core Team 2011) for the resting metabolic rate experiments. The LMER included the RMR of O_2_ as the response variable, and age, mass, sex, temperature and season as explanatory factors. We used Akaike information criteria (AIC) as a measure of the relative quality of the statistical models to remove factors that were not significantly related to RMR, and compared the full and reduced models using residual sum of squares (RSS) criteria. The final linear model contained the effects of season and temperature. We accepted P ≤ 0.05 as a statistically significant difference.

## Results

**Fig 1:**
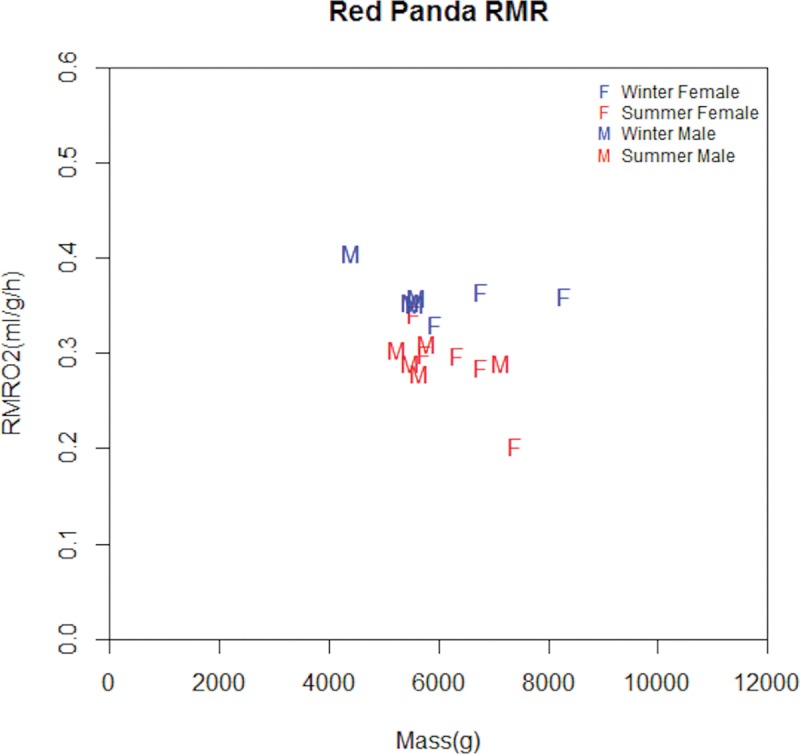
Metabolic rates of red pandas measured at the Chengdu Research Base of Giant Panda Breeding in China. Animals were at rest in a metabolic chamber at temperatures between 5.3 and 20.2°C. M represents male and F represents female. Blue represents RMR measured in winter, and red is RMR measured in summer.

Animals were asleep during the experiments. The resting metabolic rate (RMR) of the red panda ranged from 0.204 ml/h/g to 0.406 ml/h/g (Fig 1). The LMER model ANOVA indicated that there was a statistically significant effect of season (df = 1, 11; F = 28.149; P = 0.000) and temperature (df = 1, 11; F = 6.541; P = 0.027). The RMR of red pandas was 0.290 ml/h/g (n = 10, range = 0.204 ml/h/g to 0.342 ml/h/g, SEM = 0.010) (36.5 KJ/h) in summer (temperature range from 15.5°C to 20.2°C); and it was 0.361 ml/h/g (n = 7, range = 0.331 ml/h/g to 0.406 ml/h/g, SEM = 0.008) (44.3 KJ/h) in winter (temperature range from 5.3°C to 9.1°C) (Table 1). There was no difference in RMR between males and females, and no difference in RMR due to mass.

**Table 1:** Metabolic rates and RQs of red pandas measured in a metabolic chamber at the Chengdu Research Base of Giant Panda Breeding in Chengdu, China.

Animal Number	Sex	Mass (g)	Mean Temperature (C)	RMR CO_2_ ml/g/h	RMR O_2_ ml/g/h	RQ
1	M	5490	19.0	0.224	0.290	0.77
2	M	5660	18.2	0.228	0.279	0.82
3	F	5540	19.9	0.231	0.342	0.68
4	F	5750	20.2	0.211	0.299	0.71
5	M	5260	18.5	0.306	0.304	1.01
6	F	6320	15.9	0.235	0.299	0.79
7	M	7150	17.3	0.238	0.290	0.82
8	F	7380	15.5	0.191	0.204	0.94
9	F	6780	16.3	0.199	0.286	0.70
10	M	5780	17.6	0.256	0.311	0.82
11	M	5590	7.6	0.247	0.359	0.69
12	M	4420	7.9	0.354	0.406	0.87
13	F	6770	9.1	0.266	0.366	0.73
14	M	5500	8.3	0.298	0.354	0.84
15	M	5570	5.5	0.289	0.353	0.82
16	F	5920	5.3	0.293	0.331	0.88
17	F	8290	7.0	0.272	0.360	0.75

## Discussion

There was no difference in metabolic rate between male and female red pandas. Some mammals have behavioral and physiological differences between males and females that cause differences in RMR. For example, in humans, males have higher BMR and active metabolic rate than females, but female margays (*Leopardus wiedii*) have higher RMR than males [16, 17, 18]. Like many species, red panda males and females had similar RMR.

Temperature is an important factor affecting RMR. Mammals have a thermal neutral zone in which animals have a minimum RMR. Below that zone metabolic rate increases due to increased metabolic heat production. Above that zone metabolic rate increases due to a loss in the ability of the animal to cool its body temperature by behavioral and physiological means [19]. In our experiments, metabolic rates of red pandas were statistically significantly higher in winter at environmental temperatures between 5.3°C to 7.6°C than in summer at environmental temperatures of 15.5°C to 20.2°C. The lower critical temperature for the lower end of the thermal neural zone for placental mammals the size of red pandas is predicted to be about 12–17°C with actual data points for several species ranging from 4 to 25°C. The upper critical temperature is predicted to be about 28–29°C with a range of actual data points for several species ranging from 25 to 35°C [20]. Therefore, temperatures in summer season were probably within the thermal neutral zone, but winter temperatures were below the thermal neutral zone. There was no difference in activity by red pandas in the chamber during winter and summer. However, we expect that red pandas will reduce their activity to conserve heat during winter in nature. Two red pandas spent more time resting in winter than summer in the Wolong Nature Reserve, Sichuan Province, China [5]. Energy digestibility of bamboo, *Bashania spanostachya*, in the Yele Nature Reserve, Sichuan Province is low in winter and red pandas take in more energy in summer-autumn than in winter [3]. Therefore, red pandas should reduce their activity in winter to conserve energy and thermoregulation.

### Species comparisons

Contrary to our expectations based on its major diet of bamboo leaves and stalks and McNab’s data from two red pandas [10] the metabolic rate of the red panda was similar to that of other mammals of the same size. McNab’s data were well below those in our study and well below the values predicted by the regression line in Sieg et al. [11]. We compared the resting metabolic rate in ml/g/h (MR) of the red panda to those reported for 49 other mammals ranging in size from 2,010 g to 10,550 g taken from Sieg et al. [11] (Table 2). The metabolic rate of our red pandas was higher than that of some species of similar mass, such as the chimpanzee (*Pan troglodytes*), crab-eating fox (*Cerdocyon thous*), eyra cat (*Puma yagouaroundi*), and plains vizcacha (*Lagostomus maximus*); but lower than that of the raccoon (*Procyon lotor*), golden-mantled howling monkey (*Alouatta palliata*), Bornean orangutan (*Pongo pygmaeus*), culpeo (*Lycalopex culpaeus*), North American porcupine (*Erethizon dorsatum*), Guinea baboon (*Papio papio*) and lowland paca (*Cuniculus paca*). Taxonomic, physiological and environmental differences in the RMR of these species are not adequately explained by current theories [11, 21]. Diverse adaptations in metabolism have evolved in the context of physiological, biochemical, thermoregulatory and ecological constraints. Therefore, the RMR of individual species can only be understood on a species by species basis in light of these constraints

**Table 2:** Metabolic rates of 49 large mammals compiled by Sieg et al [11] and red pandas measured at the Chengdu Research Base of Giant Panda Breeding and by McNab [10].

#	Animal	Species	Mass (g)	RMR O_2_ (ml/g/h)	Log 10 (Mass)	Log 10 (MR O_2_)
1	Small-toothed palm civet	*Arctogalidia trivirgata*	2010	0.275	3.30	2.74
2	Southern viscacha	*Lagidium viscacia*	2056	0.340	3.31	2.84
3	Malagasy civet	*Fossa fossana*	2260	0.401	3.35	2.96
4	South African Springhare	*Pedetes capensis*	2300	0.341	3.36	2.89
5	Common brown lemur	*Eulemur fulvus*	2330	0.139	3.37	2.51
6	Kinkajou	*Potos flavus*	2406	0.334	3.38	2.91
7	Jamaican coney	*Geocapromys brownii*	2456	0.300	3.39	2.87
8	European wild cat	*Felis silvestris*	2618	0.180	3.42	2.67
9	Desmarest’s hutia	*Capromys pilorides*	2630	0.227	3.42	2.78
10	Groundhog	*Marmota monax*	2660	0.270	3.42	2.86
11	Red-rumped agouti	*Dasyprocta leporina*	2687	0.580	3.43	3.19
12	Tayra	*Eira barbara*	2950	0.414	3.47	3.09
13	Red fox	*Vulpes vulpes*	2965	0.488	3.47	3.16
14	Patas monkey	*Erythrocebus patas*	3000	0.213	3.48	2.81
15	Verreaux’s sifaka	*Propithecus verreauxi*	3000	0.243	3.48	2.86
16	Asian palm civet	*Paradoxurus hermaphroditus*	3160	0.241	3.50	2.88
17	Brazilian porcupine	*Coendou prehensilis*	3280	0.282	3.52	2.97
18	Margay	*Leopardus wiedii*	3550	0.283	3.55	3.00
19	White-nosed coati	*Nasua narica*	3630	0.327	3.56	3.07
20	Yellow-bellied marmot	*Marmota flaviventris*	3706	0.343	3.57	3.10
21	Collared mangabey	*Cercocebus torquatus*	3750	0.428	3.57	3.21
22	Azara’s agouti	*Dasyprocta azarae*	3849	0.490	3.59	3.28
23	South American coati	*Nasua nasua*	3850	0.260	3.59	3.00
24	Arctic fox	*Vulpes lagopus*	3933	0.458	3.59	3.26
25	African palm civet	*Nandinia binotata*	4270	0.202	3.63	2.94
26	Coypu	*Myocastor coypus*	4325	0.710	3.64	3.49
27	Mantled howler monkey	*Alouatta palliata*	4670	0.428	3.67	3.30
28	Bornean orangutan	*Pongo pygmaeus*	4970	0.305	3.70	3.18
29	Chimpanzee	*Pan troglodytes*	5020	0.280	3.70	3.15
30	Raccoon	*Procyon lotor*	5385	0.387	3.73	3.32
31	Culpeo	*Lycalopex culpaeus*	5418	0.888	3.73	3.68
32	Crab-eating fox	*Cerdocyon thous*	5614	0.272	3.75	3.18
33	Red panda	*Ailurus fulgens*	5740	0.153	3.76	2.94
34	North American porcupine	*Erethizon dorsatum*	5974	0.476	3.78	3.45
35	Red panda in this study	*Ailurus fulgens*	6069	0.290	3.78	3.25
36	Jaguarundi	*Puma yagouaroundi*	6105	0.255	3.79	3.19
37	Guinea baboon	*Papio papio*	6760	0.404	3.83	3.44
38	Plains viscacha	*Lagostomus maximus*	6804	0.234	3.83	3.20
39	Lowland paca	*Cuniculus paca*	6832	0.346	3.83	3.37
40	Black-backed jackal	*Canis mesomelas*	7720	0.505	3.89	3.59
41	Aardwolf	*Proteles cristata*	7928	0.254	3.90	3.30
42	Blue monkey	*Cercopithecus mitis*	8500	0.399	3.93	3.53
43	European otter	*Lutra lutra*	8671	0.555	3.94	3.68
44	Japanese macaque	*Macaca fuscata*	9300	0.469	3.97	3.64
45	Bobcat	*Lynx rufus*	9400	0.449	3.97	3.63
46	Olive baboon	*Papio anubis*	9500	0.311	3.98	3.47
47	Raccoon dog	*Nyctereutes procyonoides*	9800	0.409	3.99	3.60
48	Serval	*Leptailurus serval*	10120	0.329	4.01	3.52
49	Coyote	*Canis latrans*	10171	0.358	4.01	3.56
50	Mantled guereza	*Colobus guereza*	10450	0.285	4.02	3.47
51	Gray wolf	*Canis lupus*	10550	0.375	4.02	3.60

### Metabolic scaling

**Fig 2:**
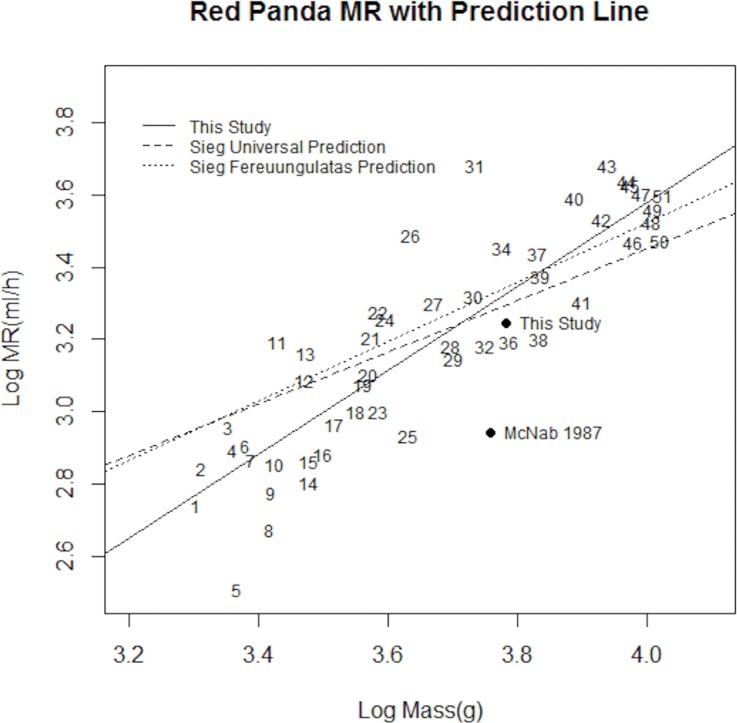
Relationship between body mass and metabolic rate in red pandas and 49 other large mammals (Table 2). Regression lines for all mammals and for Ferreuungulate mammals are from Sieg et al. [11]. Solid line is regression line calculated by us with the addition of the new red panda data.

We plotted the MRs of the red panda and the 49 other similar size mammals from Sieg et al. [11] (Fig 2). The regression line through those data (log_10_ (MR) = 1.1641 Log_10_ (Mass) - 1.0771, r^2^ = 0.74; P = 0.000) was different from that of Sieg et al. for carnivores/ungulates/pangolins (Fereuungulata) [22], [23]. The new regression line was steeper than both the Fereuungulata and universal regression lines, which were calculated from all 695 mammals in their data set [11]. That supports their conclusion that phylogenetic relationships affect the body size- metabolic rate regression and that there is not a single universal metabolic rate-body mass scaling relationship in mammals [11]. In addition, small mammals and large mammals may have different scaling relationships [21, 24].

The data sets used to calculate MR vs. body mass regressions typically have more small size mammals than large size mammals and metabolic scaling is steeper in large than in small mammals. In addition, a combination of phylogenetic relationships and physiological factors affect the metabolic rate of individual species and no one predictive line can account for all variation in the body size-metabolism relationship among mammals [21]. More research should focus on RMR variation within and among species. There is considerable variability in RMR between individuals of the same species in an experiment. Davy et al. [25] discuss the importance of repeatability and rank repeatability in behavioral experiments. We noted that there was considerable variation in RMR in our red pandas. Unfortunately we were not able to make repeated measurements on the same animals under the same conditions due to logistical limitations. We also had some red pandas that had high RQ values. We were not able to discern the reason for those results. Perhaps the animals still retained food in their gut despite having not eaten for 24 h. Future metabolic studies should determine the repeatability of RMR within an individual and the rank repeatability of RMR between individuals within a test group. Such measurements will help to refine our estimates of RMR vs. body mass for a given species and perhaps reduce the inter- specific variation in in those estimates.

### Conservation implications

The metabolism of the red panda indicates that food should not be a limiting factor in the number of red pandas that can be supported in a given nature reserve. Our resting red pandas had metabolic requirements of 876 kJ/day in summer and 1063 kJ/day in winter. In the Wolong Reserve they have an energy requirement of 3,619 kJ/day [5], which is due to their higher level of activity in nature. Based on feeding trials red pandas have an assimilation rate of 22–42% [5, 26] depending upon whether they are eating bamboo leaves or shoots. In the Wolong Reserve there is 30–40 times as much digestible energy in annual recruitment of bamboo leaves as needed for metabolism within the home range of a red panda [5]. Even if we take into account the 200% increase in energy requirements of lactating red pandas [27] there is more than enough available food in the Wolong Reserve for the number of red pandas present. In the Yele Nature Reserve, there are 1,634,529.3 kg of bamboo (*Bashania spanostachya*) per km^2^ [3], so there is much more bamboo available than red pandas can eat in a year. Red pandas share habitat with giant pandas that are major consumers of bamboo as well. Their niche requirements overlap, however, red pandas prefer sparse forests and areas far from villages and roads [28]. Therefore, absent a die off of bamboo, the metabolic requirements of the red panda will not limit its numbers in nature reserves in China. It is more likely that reserve size, location and anthropogenic disturbance will be the limiting factors in the size of red panda populations in protected areas.
